# Outcomes of immediate oncoplastic surgery and adjuvant radiotherapy in breast cancer patients

**DOI:** 10.1186/s12885-019-6104-4

**Published:** 2019-09-11

**Authors:** Kai Joachim Borm, Christine Schönknecht, Andrea Nestler, Markus Oechsner, Birgit Waschulzik, Stephanie Elisabeth Combs, Stefan Münch, Markus Niemeyer, Marciana Nona Duma

**Affiliations:** 1 Department of Radiation Oncology, Faculty of Medicine, Technical University Munich (TUM), Medical School, Klinikum rechts der Isar, Ismaninger Strasse 22, 81675 Munich, Germany; 2 Institute of Medical Informatics, Statistics and Epidemiology, Technical University Munich (TUM), Ismaninger Strasse 22, 81675 Munich, Germany; 3 Deutsches Konsortium für Translationale Krebsforschung (DKTK)-Partner Site Munich, Munich, Germany; 4 Institute of Radiation Medicine (IRM), Helmholtz Zentrum München, Ingolstädter Landstraße 1, Oberschleißheim, Germany; 5 Department of Obstetrics and Gynecology, Technical University Munich, Medical School, Klinikum rechts der Isar, Ismaninger Strasse 22, 81675 Munich, Germany; 6 Department of Radiation Oncology, University Hospital Jena, Bachstraße 18, 07743 Jena, Germany

**Keywords:** Local control rate, Oncoplastic surgery, Adjuvant radiotherapy, Boost definition, Breast conserving therapy

## Abstract

**Background:**

Oncoplastic surgery techniques lead to a rearrangement of the breast tissue and impede target definition during adjuvant radiotherapy (RT). The aim of this study was to assess local control rates after immediate oncoplastic surgery and adjuvant RT.

**Methods:**

This study comprises 965 patients who underwent breast-conserving therapy and adjuvant RT between 01/2000 and 12/2005. 288 patients received immediate oncoplastic surgery (ONC) and 677 patients breast-conserving surgery only (NONC). All patients were treated with adjuvant external tangential-beam RT (total dose: 50/50.4 Gy; fraction dose 1.8/2.0 Gy). An additional boost dose of 10–16 Gy to the primary tumor bed was given in 900 cases (93.3%). Local control rates (LCR), Progression free survival (PFS) and overall survival (OS) were assessed retrospectively after a median follow-up period of 67 (Q25-Q75: 51–84) months.

**Results:**

No significant difference was found between ONC and NONC in regard to LCR (5-yr: ONC 96.8% vs. NONC 95.3%; *p* = 0.25). This held also true for PFS (5-yr: ONC 92.1% vs. NONC 89.3%; *p* = 0.09) and OS (5-yr: ONC 96.0% vs. NONC 94.8%; *p* = 0.53). On univariate analyses G2–3 (*p* = 0.04), a younger age (*p* = 0.01), T-stage (*p* < 0.01) lymph node involvement (*p* < 0.01) as well as triple negative tumors (*p* < 0.01) were identified as risk factors for local recurrence. In a propensity score stratified Cox-regression model no significant impact of oncoplastic surgery on local control rate was found (HR: 2.05, 95% CI [0.93; 4.51], *p* = 0.08).

**Conclusion:**

Immediate oncoplastic surgery seems not to affect the effectiveness of adjuvant whole breast RT on local control rates in breast cancer patients.

## Background

Advances in the multimodal therapy of breast cancer have constantly improved the outcome in last decades. According to current studies an overall survival (OS) of over 90% and a local control rate (LCR) of over 95% can be achieved after 5 years when treatment modalities such as surgery, radiotherapy (RT) and systemic therapy are combined [1, 2]. However, most patients suffer from cosmetic impairment after surgical resection of the breast. Wide excision is shown to be a predictive factor for poor cosmetic results and a decrease of quality of life [3]. Thus, minimally invasive procedures and surgical reconstruction techniques are becoming increasingly important in breast cancer treatment. Surgical reconstruction techniques comprise breast reshaping techniques (e.g. Rotation flaps, reduction mammoplasty) and volume replacement procedures (latissimus dorsi flap, transverse rectus abdominis flap, expander) [4]. Due to potential donor site morbidity associated with volume replacement techniques, volume displacement techniques are the preferred method for concomitant reconstructive surgery during breast conservative surgery (BCS) [5]. These techniques lead to a rearrangement of the breast tissue and increase the anatomical changes caused by the surgical procedure. There is good evidence that most ipsilateral recurrences occur close to the site of the primary tumor, and that they arise often from residual tumor cells in tumor bed [6]. Adjuvant RT is performed to inactivate these residual tumor cells and thus to increase local control rates [2]. Immediate volume displacement procedures after tumor resection are challenging for radiation oncologists, as these surgical procedures potentially change the site of the primary tumor bed and the definition of the boost target volume might be impaired.

There is a lack of evidence in literature regarding the effectiveness of adjuvant RT on local control rates after breast conserving therapy and immediate volume displacement techniques. Previous studies addressing the oncological outcome after immediate breast reshaping neglected important factors related to the RT treatment [3, 5, 7–9]. Thus, it remains difficult to estimate the impact of reconstruction techniques on the success of adjuvant RT. This study was performed to evaluate the oncologic outcome after breast conserving therapy and immediate volume displacement reconstruction in a large patient collective taking RT as an important factor of local control in breast cancer into account.

## Methods

### Patients

**Fig. 1:**
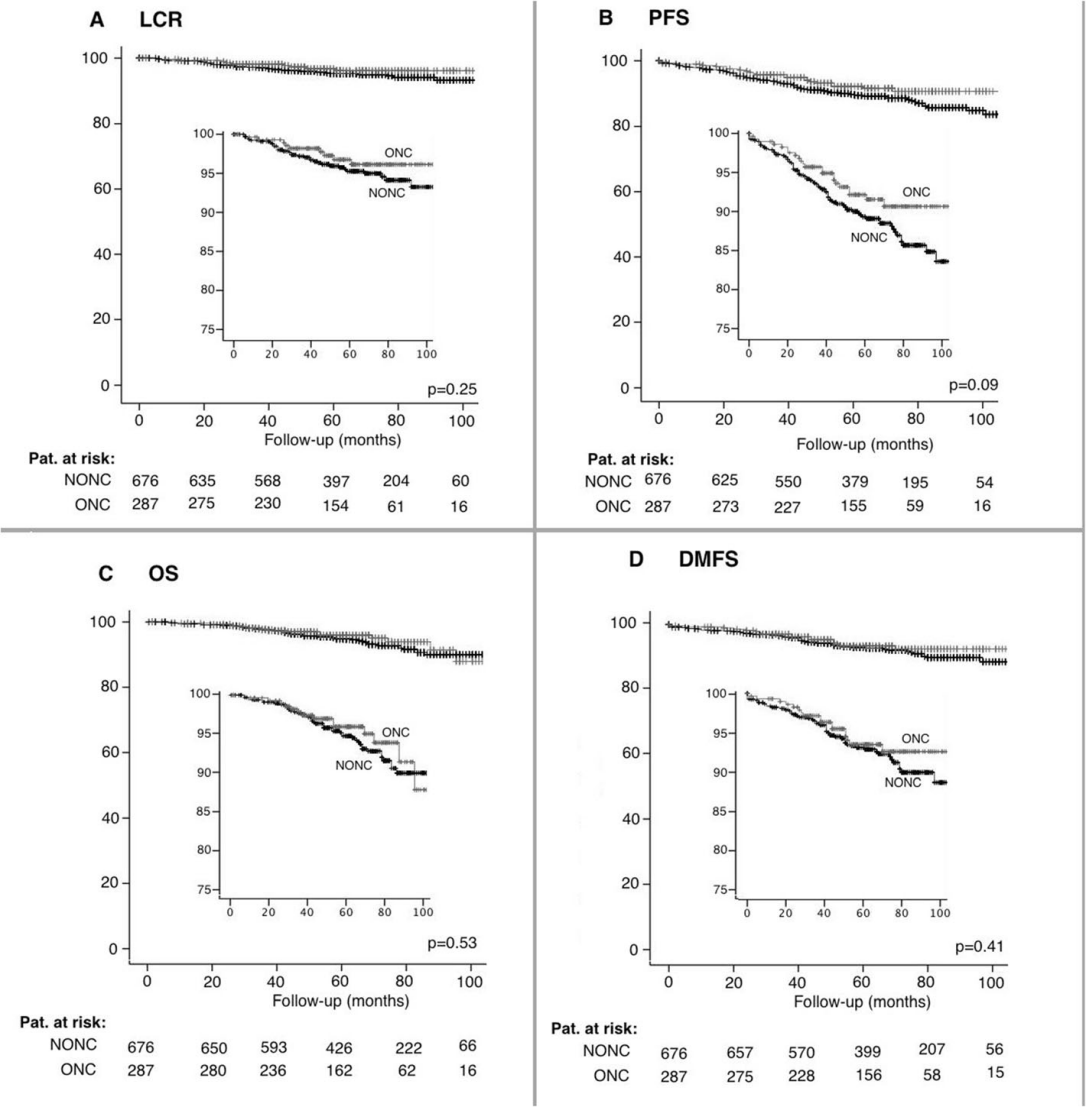
Adjuvant radiotherapy after oncoplastic surgery (bilateral reduction mammoplasty). **a**) axial plane **b**) coronar plane **c**) saggital plane right breast: breast with dose distribution and target volumes after reduction mammoplasty. Left breast: contralateral breast after reduction mammoplasty **d**) preoperative design of reduction mammoplasty **e**) preoperative design of rotation flap

This study comprises 965 patients who underwent adjuvant RT between 01/2000 and 12/2005 at the department of radiation oncology, Klinikum rechts der Isar, Munich. All patients received BCS and had no distant metastases at the time of diagnosis. Patient with other malignancies in addition to breast cancer were excluded from this analysis. In 288 cases immediate oncoplastic surgery (ONC) was performed of which 265 patients received a rotation flap and 23 patients reduction mammoplasty. One patient received volume replacement (thoraco-epigastric flap). The remaining 677 patients undergoing BCS were assigned to the control group (No oncoplastic surgery; NONC). Incisions of the rotation flap include a semi-circular line (usually in the upper quadrants) and a parallel semicircular arc at the margin line of the breast. The breast tissue within this area is removed and the bordering skin and subcutaneous tissue is used as flap and elevated and rotated to fill the defect. Reduction mammoplasty combines the techniques of tumorectomy and bilateral breast reduction (Fig. 1a-c). With both methods, the tumor can be excised with wider margins [10]. The preoperative design of reduction mammoplasty and rotation flap are presented in Fig. 1 d and e. No surgical clips were placed in the tumor bed. Whether a patient was admitted to oncoplastic surgery or conventional breast conserving surgery was decided in an interdisciplinary approach based on factors such as tumor location, expected tissue defect as well as the age and the will of the patient. A total number of 550 patients (57.0%) were given Chemotherapy before (9.6%) or after (47.4%) the surgery. 741 (76.8%) patients received anti-hormonal therapy.

### Radiotherapy (RT)

All patients were treated with external tangential beam RT to the breast. Irradiation was delivered using high-energy (6MV) linear accelerators (Megatron KD2 or MX2, Siemens, Munich, Germany). 725 (75.1%) patients received a two-dimensional planned RT, 240 (24.9%) of the patients underwent three-dimensional CT treatment planning (Fig. 1). Patients were simulated in supine position in treatment position with both arms abducted overhead. For 2D treatment planning, treatment fields were arranged by clinical examination of the patient combined with fluoroscopic 2D simulation. Superior treatment borders were set 2 cm beyond the palpable breast and 2 cm inferior the inframammary fold. The medial and lateral and medial borders were placed at the midsternal and midaxillary line, respectively. For 3D-CT based planning the target volume and organs at risk were contoured according to the RTOG guidelines [11]. All plans (2D- and 3D-plans) consisted of 2 opposing tangential beams. If necessary, wedges were applied. During 3D-treatment planning, additional beam segments were used to improve target dose coverage and homogeneity (Intended dose distribution: PTV Dmax: < 107% of prescribed dose, Dmin > 95% of prescribed dose). A total dose of 50/50.4 Gy (fraction dose 1.8 Gy or 2.0 Gy) to the whole breast was prescribed. An additional boost dose of 10–16 Gy to the primary tumor bed was given in 900 cases (93.3%). The mean size of the boost was 51.6 ± 28.5 cm^2^. The boost was applied percutaneously with a linear accelerator as direct field with electrons or 3D planned with photons (electron boost: *n* = 873, photon boost *n* = 17, not applicable *n* = 73). The location of the tumor bed was estimated based on mammography images and tissue scars. In 98 cases, the supraclavicular lymph node levels were part of the target volume with a median prescribed dose of 46 Gy (45–60 Gy). The mean time interval between BCS and postoperative RT was 91.1 ± 63.8 days.

### Follow-up and statistical analyses

A retrospective review of the relevant patient- and treatment-related factors was performed. The median clinical follow-up period was 67 (q25-q75: 51–84) months. A radiological examination of the breasts was done at least every year (including bilateral ultrasound and mammography) during the follow-up period. The clinical endpoints evaluated in this study were overall survival, progression free survival (PFS), as well as the local control rate, measured from the date of surgery. PFS was defined as the time elapsed between surgery and tumor progression, with censoring of patients who were lost during follow-up or died to any cause during follow-up. OS was defined as time between surgery and death to any cause, with censoring of patients who were lost during follow up. Local control rate was defined as freedom of local recurrence in the treated breast, with censoring of patients who were lost during follow up or died to any cause during follow-up. The treatment groups that received oncoplastic surgery (glandular flap and reduction mammaplasty) were considered as common category for statistical analysis. One patient who received volume displacement was excluded from our analysis. Overall survival and progression-free survival curves as well as local control rates were obtained according to the Kaplan-Meier method and compared between groups using the log-rank test. To evaluate differences between the two treatment groups (reconstruction vs. no reconstruction) in regard to patient, disease and treatment characteristics *chi-squared*-tests (or Fisher’s exact tests, when more than 20% of cells have expected frequencies < 5) for categorical variables and independent samples t-tests for continuous variables were used. Propensity score stratification was applied to minimize the potential bias caused by an uneven distribution of risk factors for local failure between the treatment groups. The propensity score was calculated for every patient by a multiple logistic regression model including tumor size, nodal status, age, grading, luminal status, resection margin, treatment with trastuzumab and boost as independent variables and treatment as dependent variable. For LCR, a Cox regression model with treatment group as independent variable and propensity score quartiles as strata was fitted to the data providing a stratified hazard ratio (HR). SPSS 25 software was utilized for all analyses (SPSS Inc. Chicago, IL, USA). All tests were performed two-sided on a significance level of 5%.

## Results

Progression free survival was 90.1% after a follow-up period of 5 years. At the same time a local control rate of 95.7% and an overall survival of 95.1% were observed. During the follow-up period, local recurrence in the breast occurred in 43 cases (NONC *n* = 34, ONC *n* = 9), 11 patients developed regional lymph node metastases (NONC *n* = 7, ONC *n* = 4) and 70 patients distant metastases (NONC *n* = 53, ONC *n* = 17). Of 59 deaths (NONC *n* = 45, ONC *n* = 14) that were recorded during the follow-up period only 15 could be assigned to breast cancer specific causes.

Patients receiving immediate reconstructive surgery were significantly younger compared to patients without reconstructive surgery (55.8 ± 10.5 vs. 58.2 ± 10.4 years; *p* < 0.01). Furthermore, tumors in the ONC group were significantly larger (Ø17.6 ± 10.5 cm^3^ vs. Ø15.4 ± 9.5 cm^3^; *p* < 0.01), hence more patients met the criteria for a stage T2 tumor as compared to the control group. Patients with oncoplastic surgery had significantly more often involvement of the regional lymph nodes (33.3% vs. 24.4%; *p* < 0.01). The proportion of patients with positive margins was not statistically significant different among the two therapy groups (1.3% vs. 1.7%; *p* = 0.31). HER-2/neu proto-oncogene was amplified more frequently in the reconstruction group (14.6%) compared to the control group (10.6%; *p* = 0.07). While there was no significant difference in terms of the progesterone hormone receptor status (*p* = 0.74), a higher percentage of estrogen positive tumors (*p* = 0.03) was recorded in the control group. The characteristics of both groups are summarized in Table 1.

**Table 1:** Patients characteristics. Comparison between the oncoplastic surgery cohort (ONC) and the non-oncoplastic sugery cohort (NONC)

Variable	*N*	ONC	*N*	NONC	*p* value
N. group		288		677	
Age					
Mean ± SD	288 (100%)	55.8 ± 10.5	677 (100%)	58.2 ± 10.4	0.001
Grading					
G1	273 (94.8%)	21 (7.7%)	634 (93.6%)	87 (13.7%)	< 0.001
G2		129 (47.1%)		340 (53.5%)	
G3/4		124 (45.0%)		209 (32.9%)	
T stage					
yT0	288 (100%)	4 (1.4%)	677 (100%)	21 (3.1%)	0.012*
CIS		12 (4.2%)		36 (5.3%)	
Tmic		2 (0.7%)		2 (0.3%)	
T1a		3 (1.0%)		44 (6.5%)	
T1b		48 (16.7%)		110 (16.2%)	
T1c		135 (46.9%)		316 (46.7%)	
T2		77 (26.7%)		145 (21.4%)	
T3		4 (1.4%)		1 (0.1%)	
T4		3 (1.0%)		2 (0.3%)	
N stage					
N0	288 (100%)	192 (66.7%)	677 (100%)	512 (75.6%)	0.013
N1		62 (21.5%)		120 (17.7%)	
N2		23 (8.0%)		33 (4.9%)	
N3		11 (3.8%)		12 (1.8%)	
Margins					
Negative	288 (100%)	283 (98.3%)	677 (100%)	668 (98.7%)	0.307
Positive		5 (0.7%)		9 (1.3%)	
Estrogen receptor status					
Negative	286 (99.3%)	58 (20.3%)	661 (97.6%)	180 (27.2%)	0.024
Positive		228 (79.7%)		481 (72.8%)	
Progesteron receptor status					
Negative	285 (99.0%)	108 (38.9%)	661 (97.6%)	243 (36.8%)	0.741
Positive		177 (62.1%)		481 (63.2%)	
Her2neu					
Negative	288 (100%)	246 (85.4%)	677 (100%)	605 (89.4%)	0.071
Positive		42 (14.6%)		71 (10.6%)	
Site					
Right breast	288 (100%)	154 (53.5%)	677 (100%)	313 (46.2%)	0.039
Left breast		134 (46.5%)		364 (53.8%)	
Neoadjuvant Chemotherapy					
No	288 (100%)	267 (92.7%)	677 (100%)	605 (89.4%)	0.107
Yes		21 (7.3%)		72 (10.6%)	
Adjuvant Chemotherapy					
No	288 (100%)	124 (43.1%)	677 (100%)	384 (56.7%)	< 0.001
Yes		164 (56.9%)		293 (43.3%)	
Hormone therapy					
No	288 (100%)	53 (18.4%)	677 (100%)	171 (25.3%)	0.021
Yes		235 (81.6%)		506 (74.7%)	
Trastuzumab					
No	283 (98.3%)	275 (97.2%)	675 (99.7%)	660 (81.5%)	0.577
Yes		8 (2.8%)		15 (2.2%)	

*N*=number of analyzed patients, (%).(*Fishers exact test)

With regard to RT, the total dose prescription in both cohorts was very similar (60.0 ± 5.6 Gy vs. 60.0 ± 4.7 Gy; *p* = 0.93). Nevertheless, boost irradiation was omitted more often in the oncoplastic group (ONC 9.0% vs. NONC 5.8%; *p* = 0.06). Regional lymph node irradiation was more frequently part of the treatment in the oncoplastic surgery group (14.5% vs. 9.7%; *p* = 0.02). Furthermore, it was found that the time interval between surgery and RT was significantly longer in patients who received oncoplastic surgery (85.9 ± 61.2 d vs. 105 ± 68.0 d; *p* < 0.01). In patients without adjuvant chemotherapy the time interval after oncoplastic surgery was only slightly prolonged (NONC: 64.5 ± 42.8 vs. ONC 68.4 ± 41.9; *p* = 0.43).

**Fig. 2:**
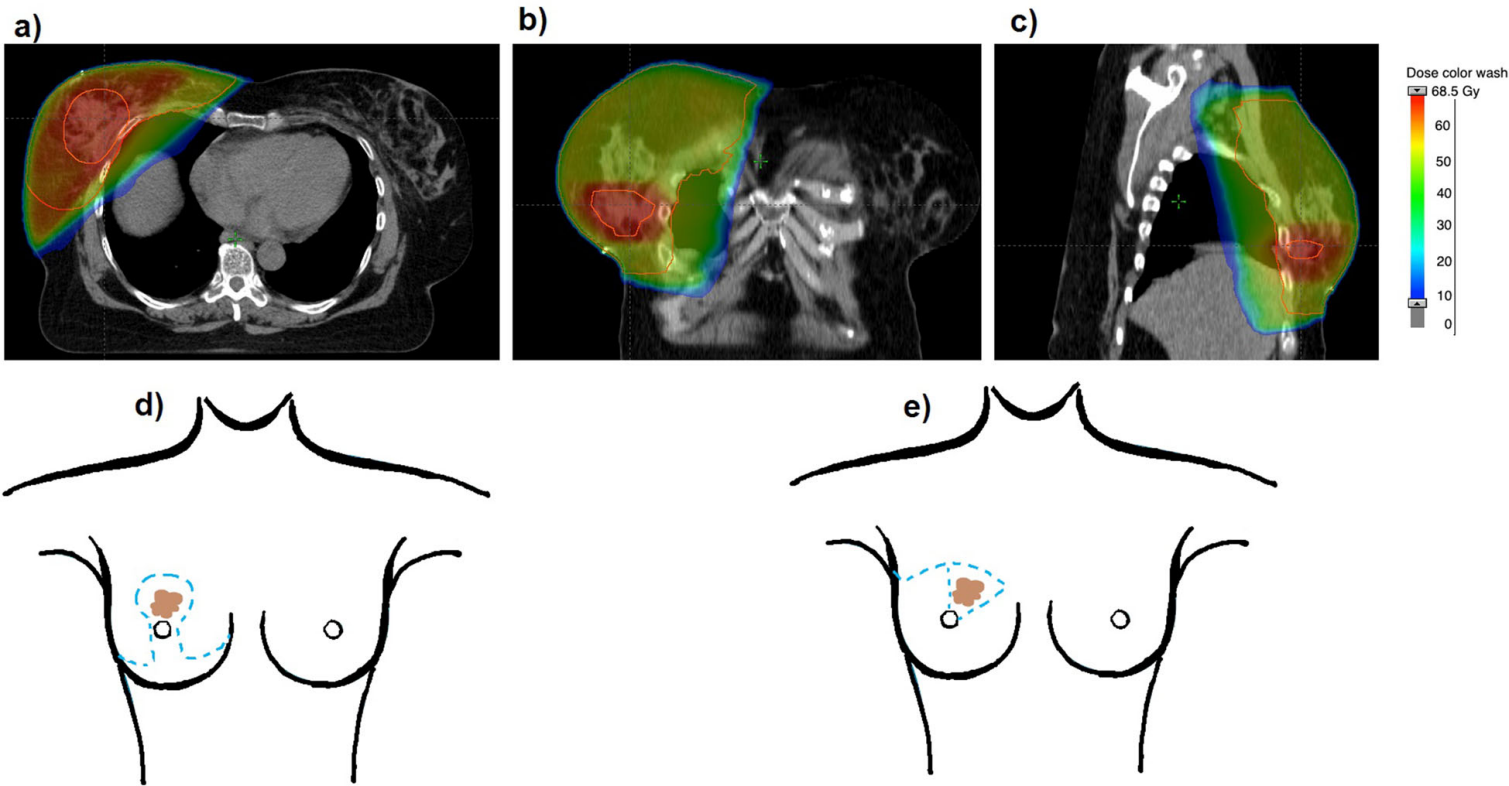
Kaplan-Meyer Curves. Oncological outcome after oncoplastic surgery (ONC) and non-oncoplastic surgery (NONC). **a**) Local control rate **b**) Progression free survival **c**) Overall survival **d**) Distant metastastases free survival

The scheduled 5-year probability of LCR was slightly higher in the oncoplastic surgery group with a value of 96.8% as compared to the control group (95.3%). However, no significant difference between LCR was observed in the log-rank test (HR = 1.54; 95% CI [0.74; 3.20]; *p* = 0.25). This held also true for PFS (5-yr: ONC 92.1% vs. NONC 89.3%; HR = 1.51; 95% CI [0.94; 2.42]; *p* = 0.09) and OS (ONC: 96.0% NONC 94.8%; HR = 1.21, 95% CI [0.67; 2.21], *p* = 0.53). The 5-yr distant metastases free survival (DMFS) was 92.7% (ONC) vs. 93.0% (NONC), HR = 1.26, 95% CI [0.73; 2.17], *p* = 0.41). Kaplan-Mayer curves regarding LCR, PFS, OS and DMFS can be found in Fig. 2. On univariate analyses G2–3 (*p* = 0.03), a younger age (*p* = 0.01), higher T-stage (*p* < 0.01), lymph node involvement (*p* < 0.01) as well as triple negative tumors (*p* < 0.01) were identified as risk factors for local recurrence. Patients receiving no boost had a similar local control rate after 5 years compared to patients without boost (95.2% vs. 95.4%; *p* = 0.57). No association between a longer interval between surgery and radiotherapy and local control rate was found (*p* = 0.26).

Similar to univariate analyses no significant impact of the surgery technique on local control rates was found in the propensity score stratified Cox regression model. There was a trend toward a better local control rate in the oncoplastic surgery group. The stratified Hazard ratio for local recurrence was 2.05 (95% CI 0.93; 4.51; *p* = 0.08).

## Discussion

Our results confirm immediate oncoplastic surgery as a safe treatment technique in breast cancer patients. Similar local control rates were observed in univariate and multiple analyses after immediate oncoplastic surgery and breast conserving surgery only. According to our results it can be assumed that the rearrangement of tissue during oncoplastic surgery does not affect the efficacy of adjuvant irradiation in a relevant way.

The oncologic outcome in our collective is in good accordance with the existing data regarding adjuvant irradiation after breast conserving therapy. The local recurrence rate after breast conserving therapy and adjuvant irradiation in a large meta-analysis by the EBCTG [2] including 42,000 women was 7%, which is slightly higher compared to our collective. However, the reported local control rates in the included studies ranged from 2 to 12% after 5 years depending on the patients and treatment characteristics [12–14]. It is known that several factors influence the local control rates in breast cancer. In our collective a younger age, non-luminal tumors, tumor size, region nodal involvement and high-grade tumors were associated with a worse local control rate. The same factors were previously described as predictors of local recurrence following breast-conserving therapy in a study by Braunstein et al. [15] published 2017 in the breast cancer journal. The EORTC boost vs. no boost trail showed that boost irradiation reduces the risk for local recurrence from 13 to 9% in patients treated with breast-conserving surgery (Hazard ratio 0.65) [16]. In our collective boost application had no significant impact on the local control rate. This is comprehensible, as patients who did not received a boost had smaller tumors, favorable tumor biology and negative resection margins.

De Lorenzi et al. [8] published in 2015 the largest available data regarding oncoplastic surgery with subsequent RT so far. The authors assessed the safety of oncoplastic surgery for invasive primary breast cancer by comparing the oncologic outcome in 454 patients who underwent immediate reconstructive surgery to a group with conventional breast conserving therapy. Overall survival, disease free survival and local control rates were similar in both groups. However, the rate of local recurrence after 5 years was higher in the oncoplastic surgery group (3.2% vs. 1.8%; *p* = 0.07). This amplifies the assumption that the effectiveness of RT may be limited by oncoplastic surgery due to difficulties in determining the target boost volume. The oncoplastic surgery techniques included in their study (glandular reconstructions including local and loco regional flaps as well as reduction mammaplasty) were similar to our study. Corresponding to the results of Lorenzi, no significant difference in local control rates was found in our study. Nevertheless, contrary to the findings of De Lorenzi et al., our data indicates a moderate, however insignificant, increase of local control rates after oncoplastic surgery. Given the non-randomized nature of the studies, the differences seen in both studies can be interpreted as clinically insignificant. There is evidence in literature that surgery techniques, which provide a wide local excision, are linked to better local relapse rates [7, 17]. Wider tumor resection during oncoplastic surgery compared to standard lumpectomy potentially accounts for the similar outcome after oncoplastic surgery even though boost irradiation is impeded by rearrangement of the tissue.

A previous meta-analysis showed that a prolonged time interval between surgery and radiation is associated with a higher rate of local relapse [18]. According to our results the time interval between surgery and RT (in patients without adjuvant chemotherapy) was only slightly prolonged after oncoplastic surgery and no impact on local recurrence rate was found. This is in accordance with Tenofsky et al. [9] who showed similar time intervals to radiation therapy after ONC and NONC, even though a higher incidence of non-healing wounds were observed after oncoplastic surgery.

The main limitation of this study is the retrospective design and the neglect of modern fractionation schemes (hypofractionated irradiation) and techniques (e.g. accelerated partial breast irradiation ((APBI)). This is particularly relevant as a large proportion of the patients were stage I tumors with positive hormone receptors and thus potentially eligible for APBI. Tumor bed shifts after oncoplastic surgery seem likely to be even more relevant in partial breast irradiation. Roth et al. [19] investigated accelerated partial breast irradiation (APBI) after breast-conserving oncoplastic surgery in 136 patients. Their results suggest the feasibility of APBI after oncoplastic breast-conserving surgery in selected low-risk breast cancer patients; however, special attention to target volume definition is needed. A further limitation of our study is the fact that cosmetic outcome and complication rate were not assessed and reported. We are convinced that reporting the oncologic outcome complements the current literature better, as several previous studies have focused on cosmetic outcome and side effects [7, 9]. Nevertheless, further studies on the topic should also consider cosmetic aspects in addition to oncologic outcome in order to allow a more comprehensive evaluation of radiotherapy after ONC.

Until now, whole breast irradiation remains the standard treatment modality after BCS and the impact of oncoplastic surgery on local control rates in combination with whole breast RT is still insufficient [5]. Since oncoplastic surgery has gained relevance in the last years and the principles of oncoplastic surgery have remained the same, our results have an important impact for current clinical practice and provide valuable information for both patients and radiation oncologists [20, 21].

## Conclusion

The effectiveness of adjuvant whole breast RT and boost irradiation on local control rates in breast cancer patients seem not to be affected by immediate reconstructive surgery. Nevertheless, an interdisciplinary approach is necessary to ensure the best treatment for every patient.

## Data Availability

The datasets used and/or analyzed during the current study are available from the corresponding author on reasonable request.

## Abbreviations

BCS: Breast conserving Surgery
HR: Hazard Ratio
LAT: Latissimus Dorsi Flap
LCR: Local Control Rate
NONC: No Oncoplastic Surgery
ONC: Immediate Oncoplastic Surgery
OS: Overall Survival
PFS: Progression Free Survival
RT: Radiotherapy
TRAM: Transverse Rectus Abdominus Myocutaneous
